# Supplementary material for: Nature's All‐in‐One: Multitasking Robots Inspired by Dung Beetles
Source: Adv Sci (Weinh). 2024 Oct 30;11(47):2408080. doi: 10.1002/advs.202408080 (PMC11653611; doi:10.1002/advs.202408080)
Supplement: Supplementary file 1 — Supporting Information [file ADVS-11-2408080-s001.pdf]

ADVANCED SCIENCE
Open Access

Supporting Information

for *Adv. Sci.*, DOI 10.1002/advs.202408080

Nature's All-in-One: Multitasking Robots Inspired by Dung Beetles

*Binggwong Leung, Stanislav Gorb and Poramate Manoonpong**

Supporting Information

Nature's All-in-One: Multitasking Robots Inspired by Dung Beetles

*Binggwong Leung, Stanislav Gorb, Poramate Manoonpong**

This PDF file includes:

Dung Beetle-like Robot (ALPHA).

Object-Rolling Control of Legged Robots.

Contribution of Control Mechanisms.

Robot Orientation Analysis during Ball-rolling Behavior.

Calculation of the Roll and Pitch Control Activation Percentage.

Cost of Transport of Ball-rolling Behavior.

Generalization of the Neural Control Approach for Object Rolling in a Quadruped Robot

Loco-Manipulation Skills of Dung Beetle-Inspired Robot

Terrain Roughness Ratio

Leg CPG-based Control (LCPG)

Pseudocode

Figure S1. Component and joint descriptions for the dung beetle-like robot.

Figure S2. Tripod and atypical tripod support patterns observed in the ball-rolling. behavior of dung beetles.

Figure S3. Contributions of the roll and pitch orientation control.

Figure S4. Roll control test of a stationary robot.

Figure S5. Compliant fin ray-based tarsus structure on flat and uneven terrains.

Figure S6. Probability distributions of the roll and pitch angles during ball-rolling behavior .

Figure S7. Probability distributions of the robot orientation (roll and pitch angles) during ball-rolling in various conditions.

Figure S8. Demonstration of object rolling of a quadruped robot in simulation.

Figure S9. Comparison of the space ratio (sr) of dung beetle and different robotic models transporting a spherical object.

Table S1. Weight connections and motor neuron biases of the LCPG module of each leg.

Table S2. Parameter setup of neural loco-manipulation control.

Table S3. Abbreviations and definitions.

Table S4. Terrain roughness ratio.

Additional Supporting Information for this manuscript includes the following:

Movies S1. Adaptive ball-rolling behavior of a dung beetle-like robot (https://youtu.be/ScldrZ6n5Wc).

Movies S2. Demonstration of a quadruped robot rolling a cylindrical shape object (https://www.youtube.com/watch?v=tB6kJE8yfQg).

GitHub: https://github.com/VISTEC-IST-ROBOTICS-PUBLIC/CPG-ROC-LocoManipulation.git

Supplementary Text

Dung Beetle-like Robot (ALPHA)

ALPHA[1] (Figure S1A and S1B) is a dung beetle-like robot based on the functional morphology and biomechanics of the South African dung beetle, Scarabaeus galenus. The mechanical part of the robot are made of aluminum, and the body is divided into three parts joined by backbone joints (Figure S1). The first section is the head, which connects to the second section, comprising the prothorax and two front legs. The third section is the meso-, metathorax and abdomen, which carry the middle and hind legs. As a result, the robot has six legs (L1, L2, L3, R1, R2, and R3), each with three revolute joints: body-coxa (BC), coxa-femur (CF), and femur-tibia (FT) (Figure S1A and S1B).

All 18 robot joints are XM430-W350-R Dynamixel motors. These provide position and torque feedback. An Odroid-C4 controller board with a U2D2 motor interface mounted on top of the abdomen controls the motors. It sends control signals at an update frequency of 60 Hz. Two lithium batteries mounted on the side of the body power the Dynamixel motor. The robot has dimensions of 40 × 60 × 40cm and weighs 4.7 kg. The femur and tibia are both 20 cm in length. The silicone tips of the legs are coated with a rough rubber surface. There are 18 Dynamixel motors in total, each of which provides position and torque feedback (converted from the current).

Detailed descriptions of joint configurations and mechanical elements can be found in.[1] The robot simulation model, provided as a scene in the Coppelia simulation, is available on the GitHub repository at https://github.com/VISTEC-IST-ROBOTICS-PUBLIC/CPG-ROC-LocoManipulation.git.

The dung beetle-inspired robot was designed based on the morphology of the dung beetle[1] where its front, middle, and hind legs have different orientations and structures. Interestingly, the dung beetle does not share the same limb mechanics and design in all limbs. Previous morphological studies of dung beetles[1-3] revealed that the front, middle, and hind legs are different. The front legs have spikes protruding sidewards from the tibia, while the middle and hind legs have numerous fine hairs on the inner tibia. We hypothesized that the spikes can facilitate locomotion during ball rolling, while the hairs can enhance ball attachment.

Rather than replicating the exact structures of dung beetle legs, we adopted a simplified approach (template principle[4]). By employing fin-ray (Figure S5) and soft materials, which have structurally/geometrically differences from insect tarsus, [5] we focused on mimicking the functional aspects of the spikes and hairs to enhance ball-rolling. While our study may not fully capture the biomimetic potential, it demonstrates how morphological computation, such as adjusting leg structure for support or modifying leg-substrate interactions, can improve the robot's ball-rolling performance.

Object-Rolling Control of Legged Robots

Instead of a rolling action, the simplest technique for conveying an object using an existing locomotive leg is to push or kick a spherical object. Numerous studies have explored this approach by utilizing methods such as hierarchical state machines[6-8] or reinforcement learning.[9-12] However, these studies mostly considered objects that were small and light compared with the size of the robot. Another aspect of kicking is that it is a one-time impulse action on an object, which can make it difficult to control the entire trajectory of the object in an unpredictable environment.

Many studies have used reinforcement learning to train robots to perform ball-spinning or -rolling actions. For example, a hand robot has been taught to rotate a ball in its hand.[13-14] A quadruped robot platform was trained to rotate a ball while remaining motionless on the floor in an upside-down position. [15] A quadrupedal robot that can balance on a ball is the most dynamic form. [16,17] These results provide a solid foundation for the use of reinforcement learning to solve the ball-rolling motion. However, a completely functional system to emulate the dung beetle-like ball-rolling object transport motion is still lacking because these approaches only include the interaction between the legs and the ball. To successfully mimic the ball-rolling behavior inspired by dung beetles, the entire system should be modeled, including the interactions between 1) a group of front legs and the ground and 2) a group of middle and hind legs and the ball.

Currently, bio-inspired control, as with our suggested neural-based control, most closely resembles the rolling motion of dung beetles.[18-20] The implementation of control methods such as modular neural control[19] and distributed rule-based control[18] enables dung beetle-like robots to effectively execute ball-rolling behavior. Nevertheless, the evaluation of these approaches has been limited to flat terrain in the context of robot simulations.

In terms of the neural control architecture, our earlier work[19] on modular neural control allowed a simulated robot to roll a ball with a tripod gait and used body orientation feedback to adapt its movement to roll a ball uphill. The legs of the robot were all controlled using a single CPG module. In contrast, each leg of the proposed neural-based control in this study has its own LCPG-based control module. This distributed LCPG-based control architecture allows the legs to be loosely coupled, via the ROC mechanisms, resulting in an independent phase relationship

between the legs. This method can generate decoupled relationships that mimic the locomotion of dung beetles with a tripod support pattern and an atypical tripod support pattern (Figure S2).

A CPG module is primarily used to generate periodic leg movements in both previous control systems and our proposed bio-inspired neural-based control system. However, the primary contribution of this study that allows the robot to adapt to diverse settings, such as different terrains, ball properties (hard and soft), and ball weights (2 kg and 4.6 kg), is the proposed ROC module. The control concept assumes linearity in the dynamics of the rolling posture (Figure 2A) and uses linear control to address the problem. Consequently, the robot can roll a ball within a specific operational range (see Figure S6A). However, this is still far from the behavior of actual dung beetles, which are good at rolling balls in very complex environments, such as sloped terrain with a very large or heavy dung ball. Therefore, a more complex control mechanism that can handle the non-linearity of this problem should be investigated, and it should extend the capability of the robot to roll in more complex situations. In addition, dung beetles typically do not perfectly roll a ball without falling; however, with their morphology, they can rearrange themselves to return to the ball-rolling posture and then continue rolling the ball. Therefore, upgrading the morphology of the robot to be more durable toward falling might allow the robot to perform robust ball-rolling tasks and recover from falling, even if the control is not perfect. This issue is an important element to be explored in the future to enhance the robot performance such that it is more similar that of real dung beetles.

In summary, the scientific contribution of this work in the control principles includes insights into the key control functions required for sensory-motor coordination in complex, adaptive dual tasks involving locomotion and large object manipulation/transportation, referred to as loco-manipulation. One primitive function generates rhythmic motor patterns for basic locomotion, object manipulation, and their combined operation (locomotion-manipulation). The other, an additional module, focuses on balancing and adapting the robot's posture for stable ball rolling in various conditions (different ball types, weights, and terrains). These functions can be achieved through an integration of the leg central pattern generator (CPG)-based control (LCPG) and robot orientation control (ROC) with a distributed control architecture, in conjunction with the functional morphology and biomechanics of the robot. The LCPG mechanism leverages CPG and pattern formation network (PFN) submodules, while the ROC integrates roll and pitch control submodules. The LCPG module, comparable to the CPG found in insects, generates

stable coordinated leg movements.[21] The ROC module simultaneously modulates motor commands based on sensory feedback, such as body orientation. This is analogous to reflex-based control in insects.[21] These modular control mechanisms provide guiding principles for addressing complex sensory-motor coordination and control tasks in robotics. Our approach incorporates fundamental concepts of biological control, thus reflects potential neural-mechanical-structural functions and their roles in animal loco-manipulation behavior, and can also serve as a basis for future biological research on such behavior. Compared to the current state-of-the-art object-rolling control systems in legged robots, our solution enables a dung beetle-like robot to successfully roll large balls across various terrains.

Contribution of Control Mechanisms

To understand how the robot adapts its leg movement according to the perturbation, we analyzed the contribution of the roll and pitch control mechanisms in the ROC module to the ball-rolling behavior.

Figure S3A illustrates a soft ball rolling over uneven terrain. During rolling, the front legs alternately supported the body, and the robot oscillated in the roll direction. If the robot's tilt exceeded 10° in both the lateral left and right directions, the roll control will be activated and send a signal to modulate or adapt the front legs. In the blue highlighted region in Figure S3A, the robot was over-tilted to the right. As a result, the roll control outputted the right modulation, m_R, to modulate the CF and FT joints of the right front leg (R1), as shown in Figure S3A. The CF and FT joints were used to extend the leg to the ground (see Figure S3). The roll control modulated the right front leg (R1) to push against the ground, allowing the robot to tilt back and regain a stable roll angle. In addition, if the robot was tilted by greater than 10°, the roll control inhibited output of the rhythmic signals to the middle and hind legs. Consequently, the middle and hind legs were maintained in the default postures to grab the ball. This prevented the middle and hind legs from pushing the ball away and allows them to wait for the robot to regain its balance before continuing to roll the ball. The roll control test of the stationary robot is presented in Figure S4.

For the pitch control of the robot while rolling the ball, the normal ball-rolling posture of the robot was a pitch angle of 40° (Figure 2A). Figure S3B shows that the front shunting gain, which was set to 1 by default, will be reduced to below 1.0 if the robot's pitch angle exceeded 40°. Lowering the shunting gain reduced the amplitude of the BC joint movement of the front legs (the joint that regulates the step length of the front legs on the ground), causing the front legs to produce a smaller pushing force. If the front legs exerted excessive force, the robot may flip over if its pitch angle exceeds the region of stability (Figure S6A). However, if the robot's pitch angle was below 40°, the pitch control shunting gain (back shunting gain, see Figure S3B) will be reduced to below 1.0, thereby reducing the BC joint movement of the middle and hind legs (the joint that regulates the step length of the middle and hind legs on the ball). Consequently, the robot could slowly push itself onto the ball, and the pitch angle increases gradually toward the reference pitch angle. The contributions of the roll and pitch controls are

represented by the control activation percentage (Figure S3A and S3B). The pitch control always generated its output to control the movement amplitude of the BC joints, whereas if the robot was tilted too far to the left or right, the roll control was activated to assist in stabilizing the robot to return to its reference roll angle.

Figure S3B shows the rigid ball rolling across an uneven terrain in another experiment. The rigid ball was heavier than the soft ball, and thus the roll and pitch angles tended to oscillate less for each step owing to the higher inertia. As a result, roll control was activated with a lower frequency and amplitude than when rolling a soft ball. The front legs were modulated to support the robot's body based on the roll control modulation, whereas the middle and hind legs were suppressed if the robot was overtilted. Pitch control was also activated to allow the robot to slowly increase the pitch angle to the reference pitch angle. However, in this scenario, the pitch angle tended to be lower than when rolling the soft ball (indicating that the hind legs grabbed the ball at a lower position). The amplitude of the BC joints of the front legs tended to remain constant, whereas the amplitudes of the middle and hind legs varied constantly. Consequently, under these circumstances, the pitch control contributed more than during rolling of the soft ball. This may be because the surface friction of the rigid ball was lower than that of the soft ball, thus making it more difficult for the robot to return to its reference pitch angle.

The gait pattern of the robot indicates that it rolled the ball with a tripod gait. The front legs walked alternately on the ground, whereas the middle legs moved in a manner similar to that of the diagonal hind legs. Legs L2 and R3 have a similar swing and stance similarly, as do legs R2 and L3. When the robot rolled a ball in a stable area, where the roll angle was near zero, a tripod pattern was clearly visible. This can be observed during the initial phase of rigid ball rolling (Figure S3B). In contrast, the tripod gait was less pronounced when rolling the soft ball (Figure S3A) because of the tilting of the robot is caused by the perturbation. Consequently, the robot adapted its leg movements to maintain stability, resulting in a deviation of gait pattern from the tripod gait to an atypical tripod gait (Figure S2). For example, when the robot was perturbed in the roll direction, the support of the front leg varied according to the robot's orientation (Figure S3A). When the robot was tilted to the right while rolling a soft ball (Figure S3A), the stance phase was shifted from the leg L1 to the leg R1. In contrast, if the robot was perturbed in the direction of the pitch, causing it to slightly descend from the ball, the middle legs tended to

lose contact with the ball (Figure S3B), whereas the hind legs made contact with the ball more often.

Note that the nonsmooth transitions in the joint angle curves are a result of the ROC control method, which directly uses roll and pitch error signals (Equation (2) and (7) in the main manuscript) to rapidly adjust the corresponding joints (Equation (5), (6), (10), and (11) in the main manuscript). This rapid response (reflex) is beneficial for quick stabilization but can lead to abrupt movements. To improve smoothness, forward models with efference copies can be employed.[22,23] These models predict the sensory consequences (expected roll and pitch sensory feedback) based on motor commands (efference copies). By comparing the expected sensory feedback to the actual feedback, the robot can predict its state and proactively adjust its movements accordingly, resulting in smoother transitions.

Robot Orientation Analysis during Ball-rolling Behavior

Ball-rolling control allows the robot to roll a ball under a variety of conditions; however, if the perturbation is too large (e.g., too inclined or rough terrain), the robot may fall off the ball. This is similar to how a real dung beetle rolls a ball up to a certain level of perturbation. Therefore, in this section, we examine the orientation distribution in roll and pitch angles of the robot as it operates under various conditions to assess the limitations of the proposed control system.

In this study, we present the probability distribution of the robot orientation while performing ball rolling under various conditions (Figure S6). Only successful ball-rolling trials are displayed here. The orientation distribution of the robot using the best strategies from the previous section (LCPG+ROC control with FL+SM biomechanics) is shown in Figure S6A and S6B.

The robot orientation while rolling soft and rigid balls on flat and uneven terrains are shown in Figure S6A and S6B, respectively. For the soft ball (Figure S6A), the roll and pitch angle distributions of the ball rolling on uneven terrain had a wider distribution than those on flat terrain. This is because the robot tilts more easily on uneven terrain than on flat terrain. However, the distributions of the rolling behavior for a rigid ball were similar on both flat and uneven terrains (Figure S6B). This implies that the unevenness of the rigid ball (a small bump at the seam) may affect the stability of the ball-rolling behavior.

Interestingly, the distribution of soft ball rolling on flat terrain was quite round compared to the other conditions (Figure S6A). This could imply the type or direction of the external perturbation that applies to the system (Figure S7 for distributions of other conditions). For example, uneven terrain had a significant impact on the roll angle when the robot rolled the soft ball. As a result, we can see that a small portion of the distribution indicates that the robot is tilted to approximately 20° in the roll direction (Figure S6A).

The results show that ball rolling over an uneven terrain had the greatest area distribution for the ball-rolling task (Figure S6A and S6B), with an 80% success rate (Figure 2D). Consequently, if the robot tilts beyond certain boundaries, it may become unstable and fail to roll the ball. Consequently, we can assume that the boundaries of these regions represent the limits of the robot’s ability to steadily roll the ball (Figure S6A and S6B).

In summary, this study demonstrated the probability distribution of the robot's roll and pitch angles during ball-rolling. The larger the deviation in the robot orientation, the more likely it is that perturbation from the uneven terrain and unevenness of the ball will cause the robot to tilt. The limit of stable working region of the ball-rolling system can be shown by the edge of the probability distribution of the ball-rolling task over uneven terrain.

Calculation of the Roll and Pitch Control Activation Percentage

The roll control activation percentage is calculated using Equation (12). The left and right modulations ($m_L(t)$, $m_R(t)$) are used for the calculation. If the robot is tilted to one side and one of the modulation values is 0.1, the roll control activation becomes 100. The pitch control activation percentage is calculated using Equation (13). The front and back shunting gains ($sf_F(t)$, $sf_B(t)$) are used for the calculation. Because the shunting factor is set to 1 by default, the pitch control activation is equal to zero when the robot is in the stable reference rolling posture.

$$\textit{roll control activation}\% = \frac{(m_L(t)+m_R(t))}{0.1} * 100, \quad (12)$$

$$\textit{pitch control activation}\% = (2 - sf_F(t) - sf_B(t)) * 100, \quad (13)$$

Cost of Transport of Ball-rolling Behavior

The red stars indicate that the robot was unsuccessful in rolling the ball. The COT is calculated as the following equation:

$$\frac{P_{com}+P_{motors}}{mgv}, \quad (14)$$

where P_{com} is the power consumption of the computing resource (Odroid-C4), which is approximately 3 W; P_{motors} is the power consumption of 21 Dynamixel motors with a 12 V supply. The average motor current is measured using motor-current sensors. In addition. g is the gravitational acceleration (9.81 m/s^2). v is the average ball-rolling speed. m is the total weight of the robot and the ball.

Generalization of the Neural Control Approach for Object Rolling in a Quadruped Robot

Applying our control approach to a quadruped robot for rolling a round object (Figure S8) requires minor adjustments to the control parameter setup. These adjustments include:

1. The parameters of the CPG and PFN modules, such as $MI, W_{BC}, W_{CF}, W_{FT}, b_{BC}, b_{CF}, b_{FT}$, were adjusted because the PFN module defines the function mapping from joint-space movement to the task-space movement of the robot morphology. The robot has four legs (front left, front right, rear left, rear right legs), each with three revolute joints: hip, thigh, and calf. Therefore, parameters, such as W_{BC}, W_{CF}, W_{FT}, b_{BC}, b_{CF}, b_{FT}, were defined as W_{hip}, W_{thigh}, W_{calf}, b_{hip}, b_{thigh}, b_{calf} instead. They were set to: MI = 0.05, W_{hip} = 1.0, W_{thigh} = 0.1 (front and rear legs), W_{calf} = 0.0 (front leg), W_{calf} = 0.2 (rear leg), b_{hip} = 0.0, b_{thigh} = 0.0 (front leg), b_{thigh} = 1.2 (rear leg), b_{calf} = -1.5 (front leg), and b_{calf} = -0.9 (rear leg).

2. The number of motor output neurons was reduced from 18 to 12 to control 12 motors. The hip motor joints were modulated by the ROC to maintain balance.

Loco-Manipulation Skills of Dung Beetle-Inspired Robot

According to this bio-inspired design, we have addressed the loco-manipulation skills observed in the dung beetle in part. This can be determined by comparing the gait patterns between the two (see Figure 1C and E) and ball-rolling behavior (see Movie S1 in Supporting Information or https://youtu.be/ScldrZ6n5Wc).

The dung beetle can switch the functions of its legs. The two forelimbs can be used as arms for digging and as legs for normal walking and ball rolling while the four limbs are mainly used as legs for walking and as arms for ball rolling. Following these strategies, our robot also uses its forelimbs as legs for normal walking and ball rolling, while the middle and hind legs are used as legs for walking and arms for ball rolling. In the future, we will explore the use of forelimbs as arms for digging. Thus, in this study, we believe that the robot system can also present bio-inspired understanding from dung beetles. In previous study, we also demonstrated that the robot can walk on the ground and uneven terrain like a dung beetle[24] (see also a Supplementary video S2 at https://doi.org/10.1109/TCYB.2023.3249467/mm2 shown in our previous study).

Terrain Roughness Ratio

Measurements of terrain roughness relative to the size of the actual dung beetle and the robot can be described using the terrain roughness ratio ($\kappa = \frac{I_{substrate}}{I_{agent}}$) (see [25] for the introduction of the roughness ratio concept). Previous work scanned terrain height and calculated simulated terrain roughness by using the square error of the height of each point. For our case here, we practically evaluate terrain roughness ($I_{substrate}$) by using an inclinometer, which records slope angles at multiple points (20 measurements) across an experimental area. The slopes of the terrain can reflect height differences between measurement points. The inclinometer measures the slope angle at each point, and all measured values are averaged to obtain a single value representing the average slope of the terrain. This average slope is then used to estimate terrain roughness. [26] A higher average slope value indicates rougher terrain, while a lower average slope value indicates smoother terrain.

We normalize the terrain roughness using the leg length (I_{agent}) of the dung beetle or robot. A smaller ratio indicates that the agent's size is comparable to the terrain's roughness, whereas a larger ratio reflects a significant size difference between the agent and the terrain. For dung beetles, the challenge is greater than for our scaled robot because the terrain is relatively large compared to their size (see Table S4).

As shown in Table S4, for dung beetles, the terrain presents a greater challenge because the obstacles are relatively larger compared to their body size (high roughness ratio), making it harder to roll the ball smoothly. As observed in other experiments, terrains with a greater degree of similarity between the average distance of elevations and the ball radius show increasingly more difficulties during ball transportation. [27,28] In contrast, in our experiment, the robot faced less complex terrain, as the terrain's roughness is low compared to the size of the robot, simplifying the ball-rolling behavior. Note that, here, we classify the terrain roughness into three levels: low, medium, and high.

The high level of roughness corresponds to the conditions faced by the actual dung beetle ($0.001 < \kappa < 0.01,\ \kappa \approx 0.0035$ from Table S4).

The medium roughness corresponds to the conditions under which our robot performs on uneven terrain ($0.0001 < \kappa < 0.001,\ \kappa \approx 0.00018$ from Table S4).

The low roughness corresponds to the conditions under which our robot performs on flat terrain ($0.00001 < \kappa < 0.0001,\ \kappa \approx 0.00002$ from Table S4).

Given the differences between our robot's performance and the actual dung beetle's behavior, future work could expand our solution to address more complex ball-rolling behaviors, similar to those exhibited by the real dung beetle. This includes implementing the beetle’s recovery mechanism, where the beetle, when disrupted by terrain roughness or other external perturbations, can recover by climbing back onto the ball and resuming its rolling behavior.

Leg CPG-based Control (LCPG)

The CPG model of the LCPG mechanism is formed by two recurrent neurons (C_1, C_2) with a modulatory input (MI, Figure 6). The activities of the CPG neurons (O_1, O_2) are developed according to

$$O_1(t+1) = tanh\left(W_{11}O_1(t) + W_{12_m}O_2(t)\right), \tag{15}$$

$$O_2(t+1) = tanh\left(W_{22}O_2(t) + W_{21_m}O_1(t)\right), \tag{16}$$

$$W_{11}, W_{12} = 1.4, \tag{17}$$

$$W_{12_m} = 0.18 + MI, \tag{18}$$

$$W_{21_m} = -(0.18 + MI), \tag{19}$$

where W_{11}, W_{12} are the self-connection weights of O_1, O_2 and W_{12_m}, W_{21_m} are the connection weights between O_1, O_2. MI is set to 0.15, which determines the appropriate CPG frequency for the robot.

Pseudocode

The following table shows the pseudocode of the LCPG control.

Algorithms 1 Leg CPG-based Control (LCPG) $\begin{cases} Central\ pattern\ generator\ (CPG) \\ Pattern\ fromation\ network\ (PFN) \\ Motor\ neurons\ (MNs) \end{cases}$

Initialization: six LCPG module for each leg $LCPG_{0,1,2,3,4,5}$ or $LCPG_{L1,L2,L3,R1,R2,R3}$

For each $LCPG$

CPG:

Initialize variables MI, $O_1(t)$, $O_2(t)$, $W_{11,22}$, W_{12_m}, W_{21_m} based on Equation (1) − (5)

Step $LCPG_{1,3,5}$ for 70 time steps[1]

Initialize $t \leftarrow 1$

repeat

$O_1(t+1) \ \leftarrow \ tanh\left(W_{11}O_1(t) + W_{12_m}O_2(t)\right)$

$O_2(t+1) \ \leftarrow \ tanh\left(W_{22}O_2(t) + W_{21_m}O_1(t)\right)$

$t \leftarrow t+1$

Until $t > 70$

PFN:

Initialize variables i, τ

If $LCPG_{L1,R1}$ (front Legs)

$i = 1$ (to generate backward walking)

If $LCPG_{,L2,L3,R2,R3}$ (middle or hind legs)

$i = 0$ (to generate backward walking)

Initialize PFN module parameters $(P_1, \ldots, P_{12})$ based on circuit diagram Figure 6

Initialize weights connections between PFN and motor neurons W_{BC}, W_{CF}, W_{FT} (Table S1)

MNs:

Initialize biases for the motor neurons b_{BC}, b_{CF}, b_{FT} (Table S1)

Loop: Step the LCPG ($LCPG_{0,1,2,3,4,5}$) of all legs until the program is terminated, Equation (1), (2)

For each $LCPG$

While (True)

CPG:

$O_1(t+1) \ \leftarrow \ tanh\left(W_{11}O_1(t) + W_{12_m}O_2(t)\right)$

$O_2(t+1) \ \leftarrow \ tanh\left(W_{22}O_2(t) + W_{21_m}O_1(t)\right)$

PFN:

Two inputs (PC_1, PC_2) is calculated from $O_1(t)$ to get asymmetrical sawtooth signals

$PC_1 \ \leftarrow asym\_sawtooth(O_1(t)),\ PC_2 \ \leftarrow asym\_sawtooth(O_2(t))$

perform feedforward calculate of neurons in PFN module to get the output at P_{11}, P_{12}

$a_i(t+1) \leftarrow \sum_{j=1}^{n} w_{ij}o_j(t) + b_i;\ i = 1, \ldots n$, Equation (1)

MNs:

If $LCPG_{L1,R1}$ (front Legs)

$BC_{0,3} \leftarrow P_{11} \cdot W_{BC0,3} + b_{BC0,3}$

$CF_{0,3} \leftarrow P_{12} \cdot W_{BC0,3} + b_{BC0,3}$

$FT_{0,3} \leftarrow P_{12} \cdot W_{BC0,3} + b_{BC0,3}$

If $LCPG_{,L2,L3,R2,R3}$ (middle or hind legs)

$BC_{1,2,4,5} \leftarrow P_{12} \cdot W_{BC1,2,4,5} + b_{BC1,2,4,5}$

$CF_{1,2,4,5} \leftarrow P_{11} \cdot W_{BC1,2,4,5} + b_{BC1,2,4,5}$

$FT_{1,2,4,5} \leftarrow P_{11} \cdot W_{BC1,2,4,5} + b_{BC1,2,4,5}$

---

[1] $LCPG_{1,3,5}$ steps for 70 time steps to get π-radian phase difference between the output signals of $LCPG_{1,3,5}$ and $LCPG_{0,2,4}$ for tripod gait.

Algorithms 2 Robot Orientation Control (ROC) $\begin{cases} Roll\ Control\ (RC) \\ Pitch\ Control\ (PC) \end{cases}$

Initialization:

Roll Control:

Initialize variables $\phi_r, b, k_{CF}, k_{FT}$ based in Table S2

Pitch Control:

Initialize variables $\theta_r, \alpha, \delta,$ based in Table S2

Loop:

Roll Control:

Calculate the roll angle error, and modulate the front legs joint movements

$e_\phi(t) \leftarrow \phi_r - \phi_f(t)$

$m_L(t) \leftarrow ReLu(-e_\phi(t) - b),$

$m_R(t) \leftarrow ReLu(e_\phi(t) - b),$

$CF_0(t) \leftarrow CF_0(t) + k_{CF} m_L(t),$

$FT_0(t) \leftarrow FT_0(t) + k_{FT} m_L(t),$

$CF_3(t) \leftarrow CF_3(t) + k_{CF} m_R(t),$

$FT_3(t) \leftarrow FT_3(t) + k_{FT} m_R(t),$

Shunting inhibition mechanism will inhibit the movement of the middle and hind legs

If $abs(\phi_f(t)) > 10$:

$BC_{1,2,4,5} \leftarrow b_{BC1,2,4,5}$

$CF_{1,2,4,5} \leftarrow b_{BC1,2,4,5}$

$FT_{1,2,4,5} \leftarrow b_{BC1,2,4,5}$

Pitch Control:

Calculate the pitch angle error, and modulate the legs movements amplitude of all legs

$e_\theta(t) = \theta_r - \theta_f(t),$

$sf_F(t) = \alpha \cdot min(\frac{e_\theta(t)}{\delta} + 1,1) + (1 - \alpha) \cdot sf_F(t-1),$

$sf_B(t) = \alpha \cdot min(\frac{-e_\theta(t)}{\delta} + 1,1) + 1 - \alpha) \cdot sf_B(t-1),$

$BC_{0,3}(t) = BC_{0,3}(t) \cdot sf_F(t),$

$BC_{1,2,4,5}(t) = BC_{1,2,4,5}(t) \cdot sf_B(t),$

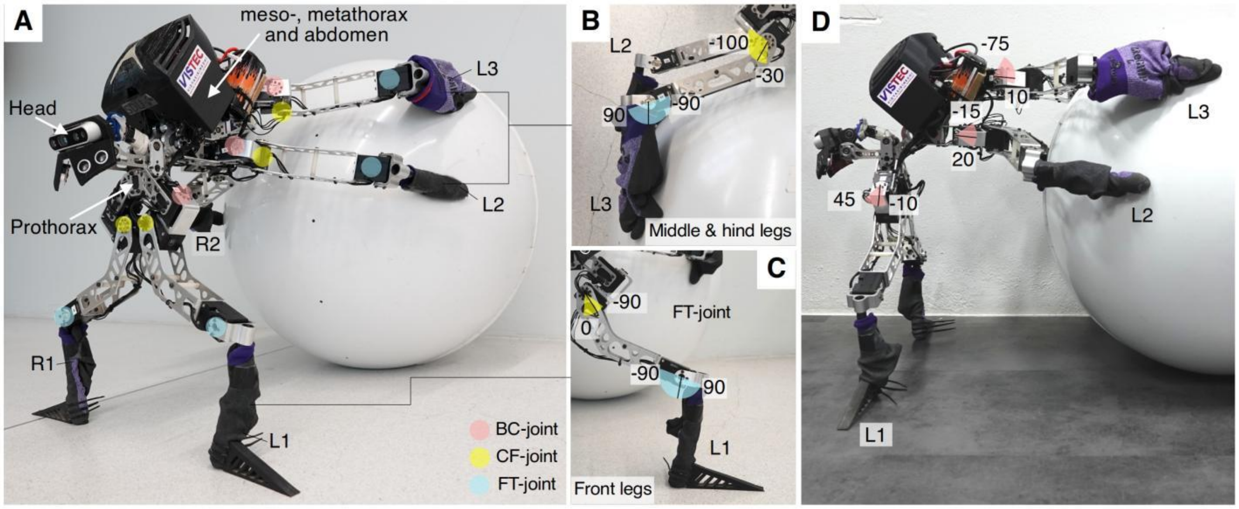

Figure S1. Component and joint descriptions for the dung beetle-like robot (ALPHA). (**A**) The robot has six legs (L1, L2, L3, R1, R2, and R3), each with three revolute joints: body-coxa (BC), coxa-femur (CF), and femur-tibia (FT). There are three parts joined by backbone joints. The first section is the head, which connects to the second section, comprising the prothorax and two front legs. The third section is the meso-, metathorax and abdomen, where the middle and hind legs are installed. (**B**) CF and FT joint ranges and the limit of the middle and hind legs. (**C**) CF and FT joint ranges and the limit of the front legs. (**D**) BC joint range and the limit of all legs.

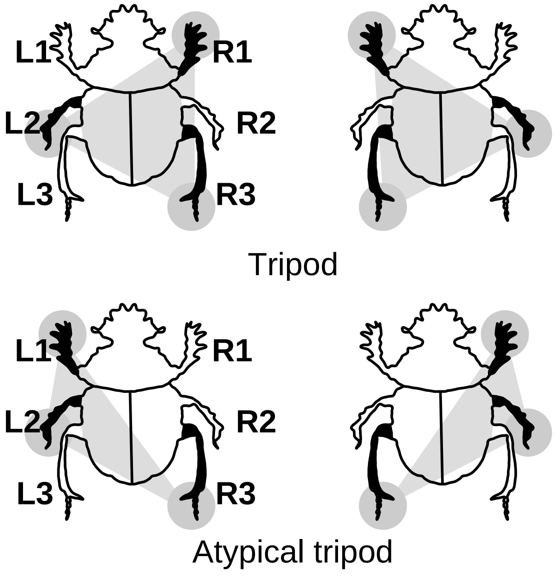

Figure S2. Tripod and atypical tripod support patterns observed in the ball-rolling behavior of dung beetles. The tripod support is characterized by the simultaneous stance of legs (left front (L1), right middle (R2), and left hind (L3)) or (right front (R1), left middle (L2), and right hind (R3)). In contrast, atypical tripod support is defined as when legs (L1, L2, and R3) or legs (R1, R2, and L3) are simultaneously in the stance phase.

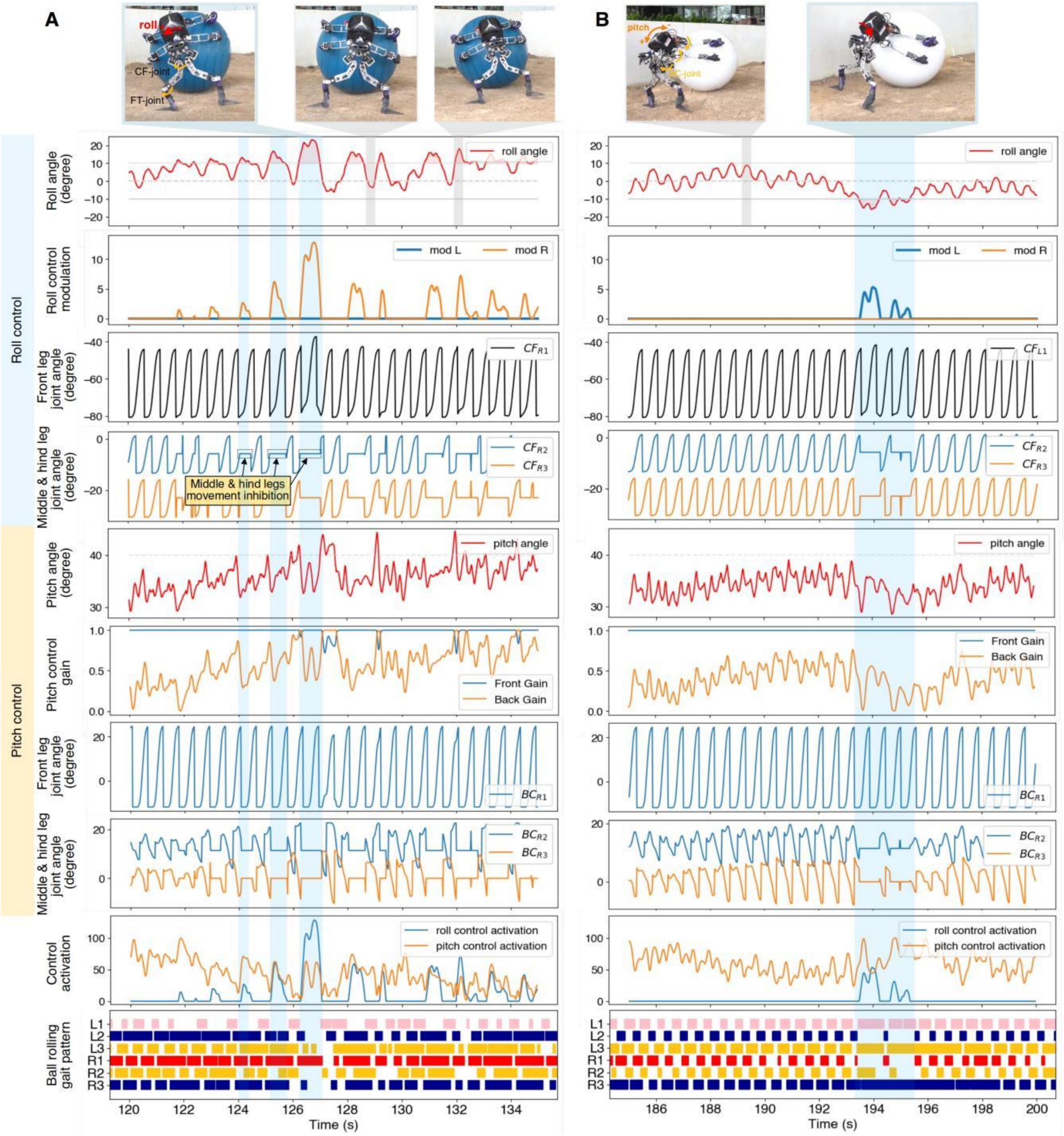

Figure S3. Contributions of the roll and pitch orientation control. (**A**) Soft ball rolling on uneven terrain. (**B**) Rigid ball rolling on uneven terrain. The roll angle, roll control modulation, and CF joint angle are used to illustrate the function of the roll control. The pitch angle, pitch control shunting gain, and BC joint angle are used to describe the function of the pitch control. The control activation percentage and gait pattern of the ball-rolling behavior are shown. For the front legs, the BC joints move horizontally along the ground, while the CF and FT joints move vertically to lift and place the leg on the ground. For the middle and hind legs, the BC joints are

used to rotate the ball, and the CF and FT joints are used to push or grasp the ball (see Material and Methods).

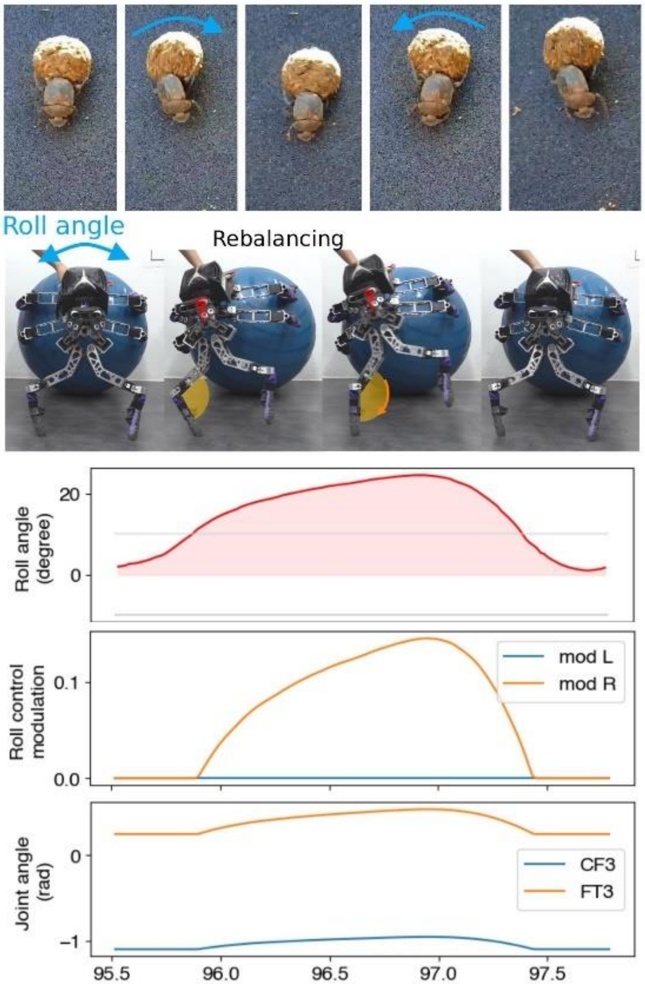

Figure S4. Roll control test of a stationary robot. Similar to a dung beetle trying to balance itself on a ball, the roll control modulates the angle of the CF and FT joints to recover balance.

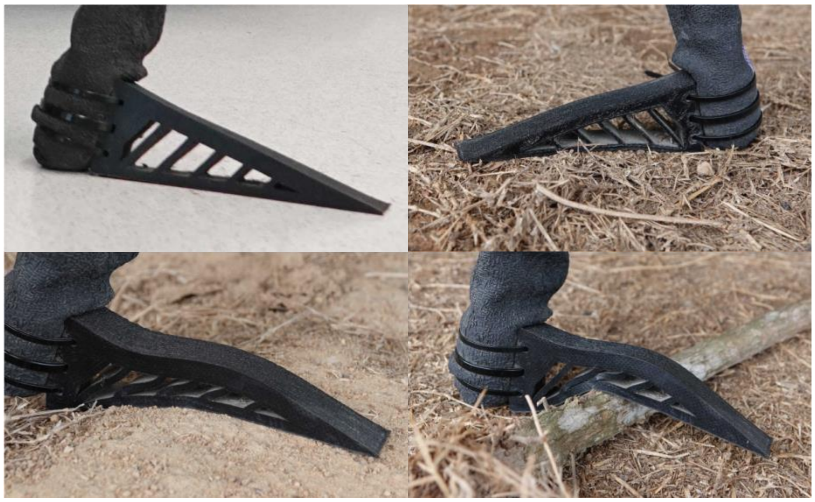

Figure S5. Compliant fin ray-based tarsus structure on flat and uneven terrains. The fin ray undergoes deformation in accordance with the curvature of the surface and the presence of an obstacle.

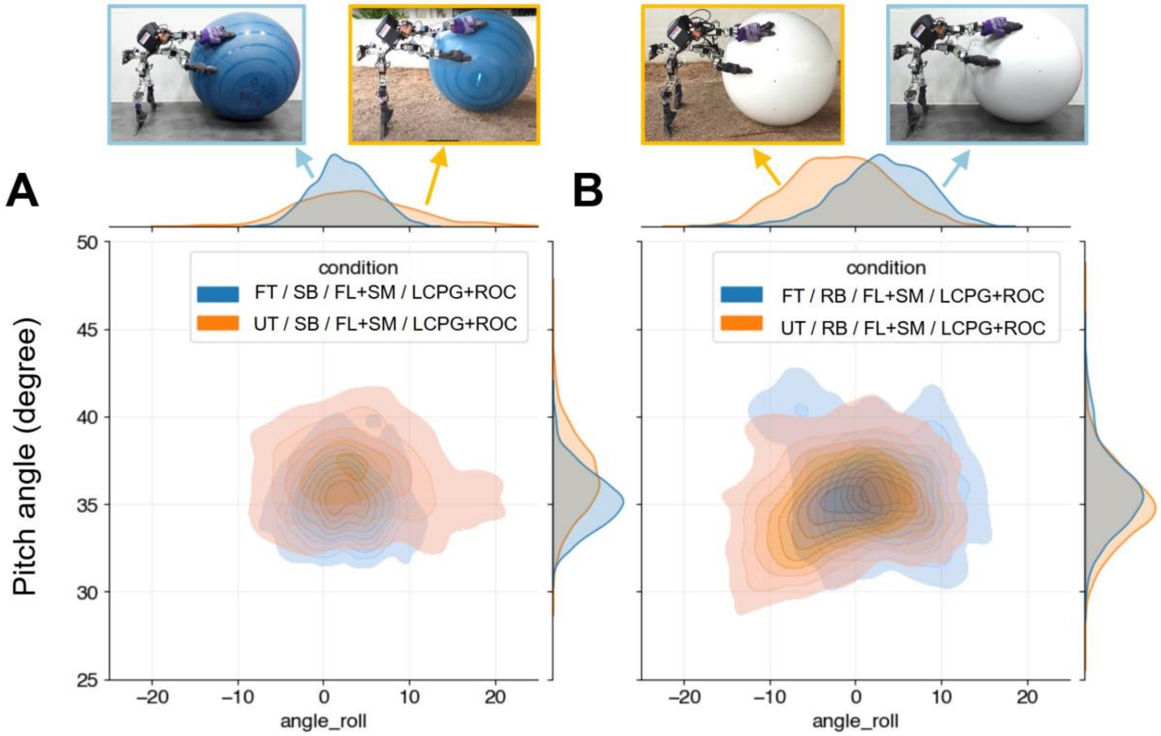

Figure S6. Probability distributions of the roll and pitch angles during ball-rolling behavior. (**A**) Soft ball on flat and uneven terrains. (**B**) Rigid ball on flat and uneven terrains.

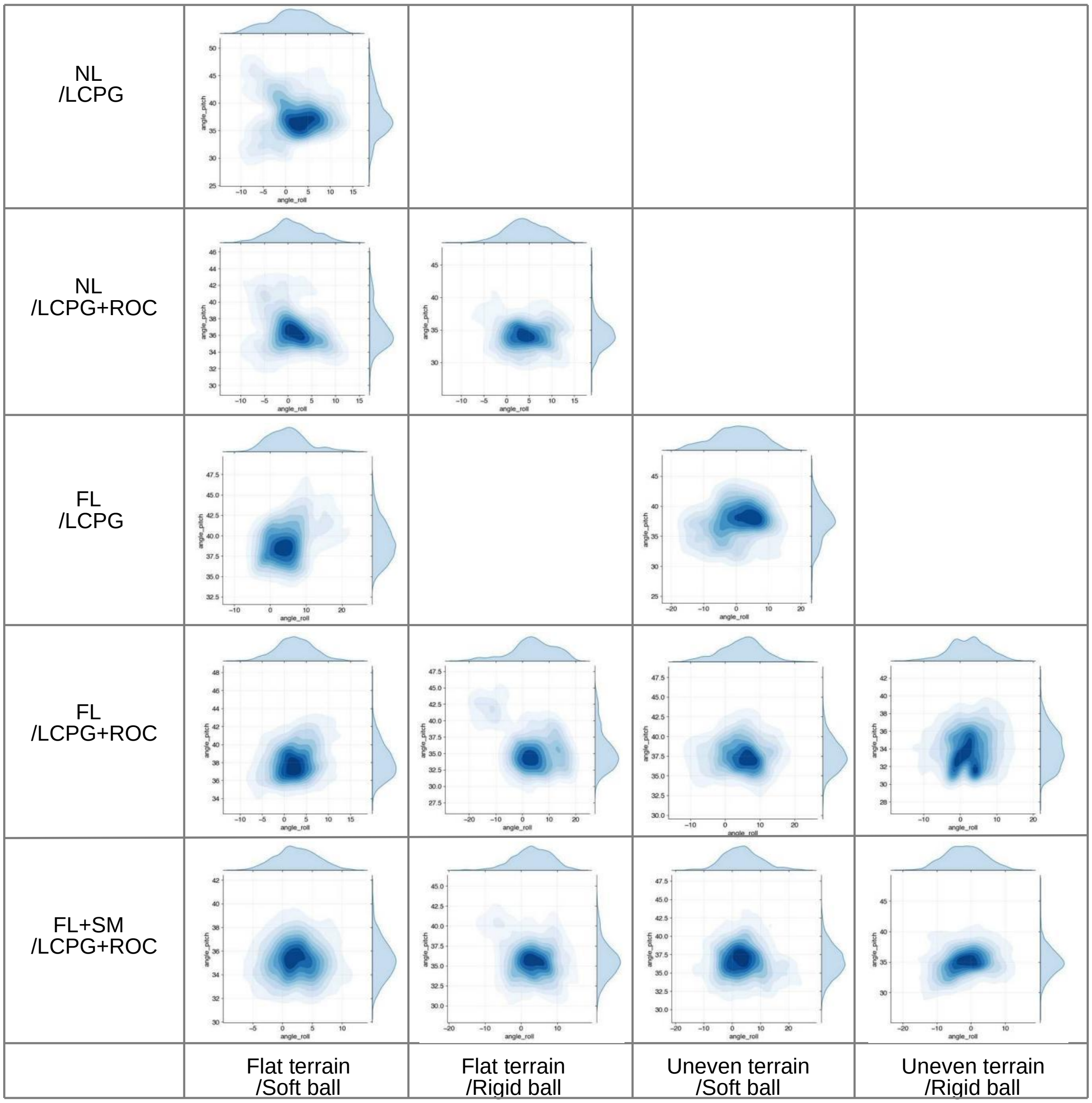

Figure S7. Probability distributions of the robot orientation (roll and pitch angles) during ball-rolling in various conditions. In different conditions, different patterns of the probability distribution can be observed. The difference may cause by different perturbation from the terrain and ball type to the robot orientation. NL/LCPG means the robot with normal legs using the LCPG module. NL/LCPG+ROC means the robot with normal legs using the LCPG and ROC modules. FL/LCPG means the robot with compliant fin ray-based tarsi attached at the front legs using the LCPG module. FL/LCPG+ROC means the robot with compliant fin ray-based tarsi attached at the front legs using the LCPG and ROC modules. FL+SM/LCPG+ROC means the

robot with compliant fin ray-based tarsi attached at the front legs with soft material at the hind legs using the LCPG and ROC modules.

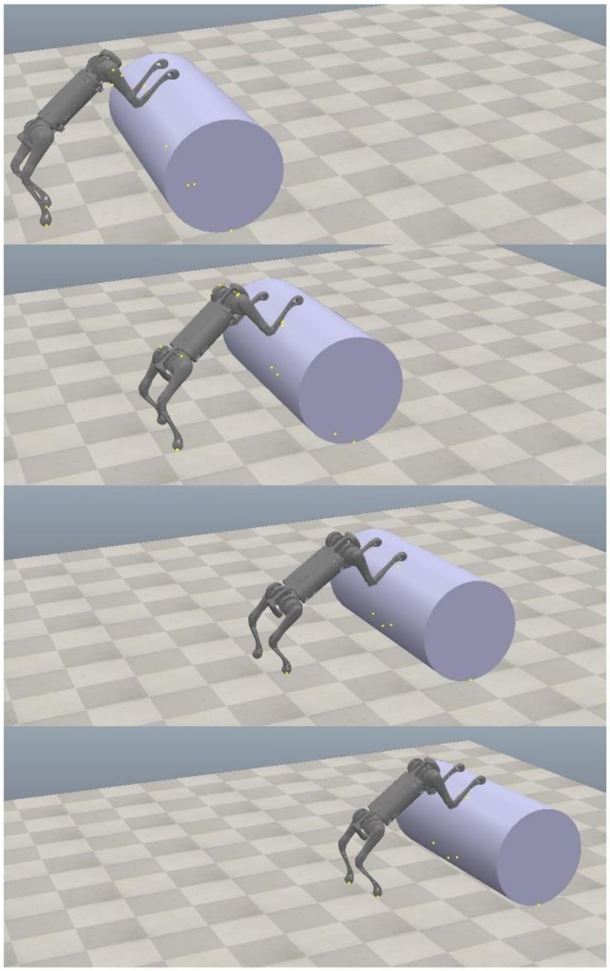

Figure S8. Demonstration of object rolling of a quadruped robot in simulation. Successful object rolling was achieved by applying the LCPG and ROC mechanisms. The robot used was the B1 quadruped robot model from Unitree, weighing 59.65 kg. The cylindrical object weighed 500 kg, had a diameter of 90 cm, and a length of 200 cm. A video of this experiment can be seen at Movie S2 or https://www.youtube.com/watch?v=tB6kJE8yfQg.

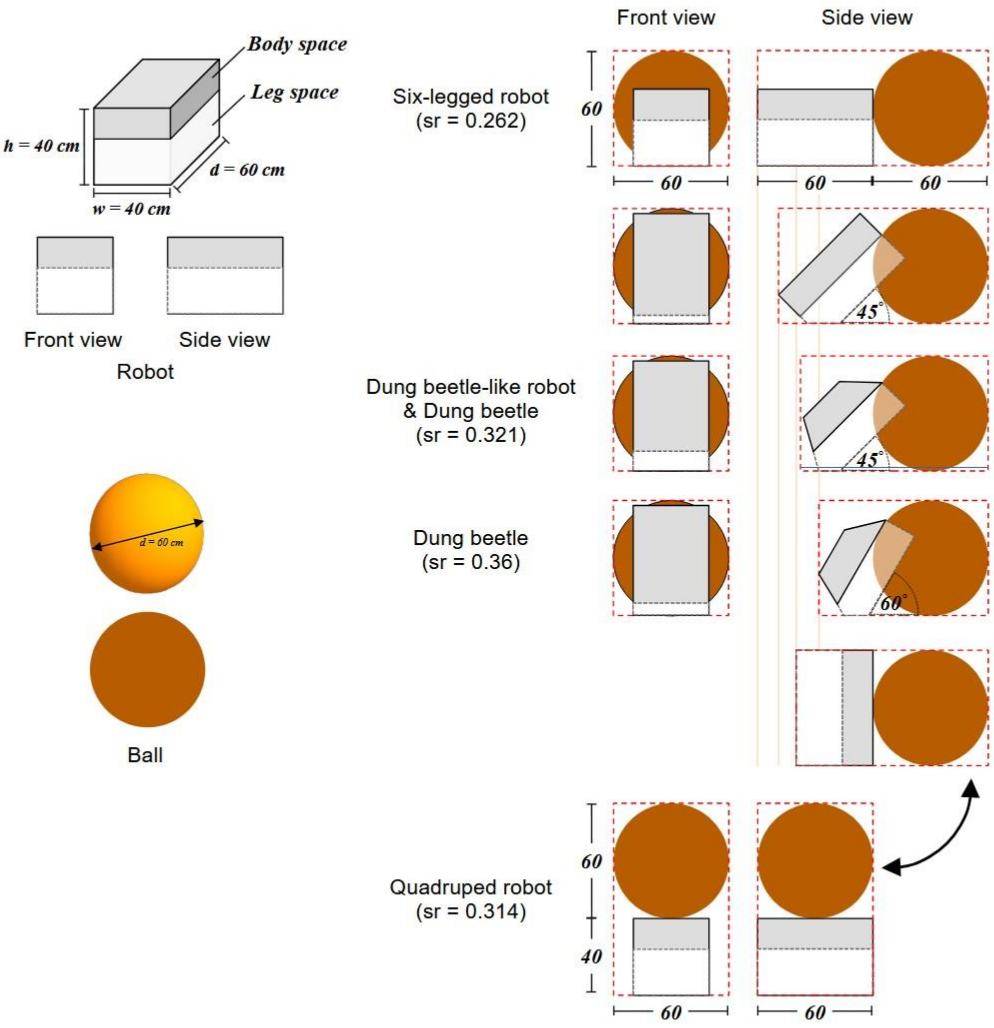

Figure S9. Comparison of space ratio (sr) for a dung beetle and various robots using different strategies to transport a spherical object. The diagram illustrates the body and leg space of each agent relative to the ball size, demonstrating task efficiency in terms of space ratio. Six-legged (sr = 0.262), quadruped (sr = 0.314), and dung beetle-like (sr = 0.321) robots are depicted with corresponding side and front views. Additionally, the dung beetle (sr = 0.36) is shown interacting with the ball at different orientations (45° and 60° angles). Notably, the total occupied space decreases when the body is tilted at a specific angle (e.g., 60°), causing the legs to overlap more with the sphere.

Table S1. Weight connections and motor neuron biases of the LCPG module of each leg. These parameters are represented in Figure 6A.

	W_{BC}	W_{CF}	W_{FT}	b_{BC}	b_{CF}	b_{FT}
L1	1.0	1.0	1.0	0.1	-1.1	0.5
L2	0.6	-0.4	0.6	0.2	-0.1	0.5
L3	0.6	-0.4	-0.6	0.0	-0.4	0.3
R1	1.0	1.0	1.0	0.1	-1.1	0.5
R2	0.6	-0.4	0.6	0.2	-0.1	0.5
R3	0.6	-0.4	-0.6	0.0	-0.4	0.3

Table S2. Parameter setup of neural loco-manipulation control.

Main control mechanisms	Neural modules	Parameters	Values	Description
Leg CPG-based control (LCPG) LCPG	Central pattern generator (CPG)	MI	0.15	Modulatory input (MI) value is related to the frequency of the CPG. The variable is referred to Equation (18), (19).
	Pattern formation network (PFN)	i	0 or 1	i is the input value for the PFN module. It is set to 0 for LCPG modules of the middle and hind legs to let these leg walk forward on the ball. It is set to 1 for LCPG modules of the front legs to let the front legs walk backward on the ground.
		τ	3	Timestep delay of the delay neuron to delay the output signals $O_1(t)$ of the CPG submodule. This parameter provides phase delay between BC, CF, and FT joints to form appropriate leg trajectory.
		W_{BC} , W_{CF}, W_{FT}	see Table S1.	Synaptic weights of the synaptic connections between the output signals of the PFN module and the motor neurons. W_{BC} , W_{CF}, W_{FT} represent the synaptic weights for BC, CF, and FT joints of front, middle, and hind legs, respectively. See Table S1 for tuned parameters of the dung beetle-inspired robot.
		b_{BC} , b_{CF}, b_{FT}		Biases value of the motor neurons. b_{BC} , b_{CF}, b_{FT} represent the biases for motor neurons of BC, CF, and FT joints of front, middle, and hind legs. See Table S1 for tuned parameters of the dung beetle-inspired robot.
Robot orientation control (ROC)	Roll control	ϕ_r	0	ϕ_r is the reference pitch angle, which is the angle at which the robot stands statically in its initial rolling posture (Figure 2A). The pitch control ensures that the robot will return to this angle for stability. The variable is referred to Equation (2).
		k_{CF}	0.1	The gains k_{CF} and k_{FT} represent the amplitude and direction of the leg's push on the ground when the robot is tilted. Both k_{CF} and k_{FT} were determined through a grid search from 0 to 1 with 0.1 intervals. The tuning process continued until the leg could extend to stably support itself when perturbed. An excessively high value could cause the leg to slip or push the ground too strong, potentially leading to the robot falling down or tilting in the opposite direction. These variables are referred to Equation (5), (6).
		k_{FT}	0.2	
		b		The roll angle bias b represents the threshold angle of the robot's roll angle to activate the leg pushing. Too low value could make the robot unstable due to the noise from IMU. Too high value means the roll control is rarely activated to provide leg support. The variable is referred to Equation (3), (4).
	Pitch control	θ_r	40	θ_r is the reference pitch angle, which is the angle at which the robot stands statically in its initial rolling posture (Figure 2A). The pitch control ensure that the robot will return back to this pitch angle for stabiltity. The variable is referred to Equation (7).
		α	0.5	α is a recurrent weight which functions similarly as a low-pass filter. First term of Equation (8), (9) represent the amplitude of the pitch error in the current timestep which is set as $\alpha = 0.5$. Whereas the second term represent the amplitude of the pitch error from previous timestep which is set as $1 - \alpha = 0.5$. α was determined using a grid search from 0 to 1 with 0.1 intervals. The tuning process continued until the leg could extend to stably support itself when perturbed.
		δ	10	δ represents a constant value for tuning the slope of the linear activation neuron. In our experiments, δ is set to 10, meaning that if the pitch angle feedback error is equal to +10, the front shunting gain will become zero. If the pitch angle feedback error is equal to -10, the back shunting gain will become zero. The variable is referred to Equation (8), (9).

Table S3. Abbreviations and definitions.

Abbreviation	Definition
CPG	Central Pattern Generator
LCPG	Leg CPG-based control
PFN	Pattern Formation Network
ROC	Robot Orientation Control
SB	blue Soft rubber fitness Ball
RB	White Rigid Ball
FT	Flat Terrain
UT	Uneven Terrain
NL	Normal biomechanical Legs covered with a textured rubber surface (Normal Leg)
FL	Front Legs with added compliant Fin-ray-based tarsi (Fin-ray Leg)
SM	Hind legs with added Soft Material.

Table. S4 Terrain roughness ratio.

Agent	Terrain	Roughness ratio (κ)
Dung beetle	Flat	0.00043
	Uneven	0.00350
Dung beetle-like Robot	Flat	0.00002
	Uneven	0.00018

References

[1] P. Billeschou, N. N. Bijma, L. B. Larsen, S. N. Gorb, J. C. Larsen, P. Manoonpong, Appl. Sci. 2020, 10, 6986.
[2] J. Ignasov, A. Kapilavai, K. Filonenko, J. C. Larsen, E. Baird, J. Hallam, S. Büsse, A. Kovalev, S. N. Gorb, L. Duggen, Artif. Life Robot. 2018, 23, 555.
[3] D. M. Linz, Y. Hu, A. P. Moczek, Proc. R. Soc. B 2019, 286, 20182427.
[4] R. J. Full, D. E. Koditschek, J. Exp. Biol. 1999, 202, 3325.
[5] Frantsevich, L., Gorb, S., Arthropod Struct. Dev. 2004, 33, 77.
[6] M. Friedmann, J. Kiener, S. Petters, D. Thomal, O. Von Stryk, H. Sakamoto, Int. J. Hum. Robot. 2008, 5, 417.
[7] C. A. Acosta-Calderon, R. E. Mohan, C. Zhou, L. Hu, P. K. Yue, H. Hu, Int. J. Hum. Robot. 2008, 5, 397.
[8] S. Behnke, J. Stuckler, Int. J. Hum. Robot. 2008, 5, 375.
[9] X. B. Peng, G. Berseth, K. Yin, M. Van De Panne, ACM Trans. Graph. 2017, 36, 1.
[10] A. Cherubini, F. Giannone, L. Iocchi, D. Nardi, P. Palamara, Robot. Auton. Syst. 2010, 58, 872.
[11] I. J. da Silva, D. H. Perico, T. P. D. Homem, R. A. da Costa Bianchi, J. Intell. Robot. Syst. 2021, 102, 69.
[12] H. Teixeira, T. Silva, M. Abreu, L. P. Reis, Humanoid Robot Kick in Motion Ability for Playing Robotic Soccer, in Proc. 2020 IEEE International Conference on Autonomous Robot Systems and Competitions (ICARSC), IEEE, New York 2020, 34.
[13] T. Pang, H. J. T. Suh, L. Yang, R. Tedrake, IEEE Trans. Robot. 2023, 39, 4691.
[14] Y. Toshimitsu, B. Forrai, B. G. Cangan, U. Steger, M. Knecht, S. Weirich, R. K. Katzschmann, Getting the Ball Rolling: Learning a Dexterous Policy for a Biomimetic Tendon-Driven Hand with Rolling Contact Joints, in Proc. 2023 IEEE-RAS 22nd International Conference on Humanoid Robots (Humanoids), IEEE, New York 2023, 1.
[15] F. Shi, T. Homberger, J. Lee, T. Miki, M. Zhao, F. Farshidian, K. Okada, M. Inaba, M. Hutter, Circus ANYmal: A Quadruped Learning Dexterous Manipulation with Its Limbs, in Proc. 2021 IEEE International Conference on Robotics and Automation (ICRA), IEEE, New York 2021, 2316.
[16] C. Yang, B. Zhang, J. Zeng, A. Agrawal, K. Sreenath, Dynamic Legged Manipulation of a Ball Through Multi-Contact Optimization, in Proc. 2020 IEEE/RSJ International Conference on Intelligent Robots and Systems (IROS), IEEE, New York 2020, 7513.
[17] Y. J. Ma, W. Liang, H. Wang, S. Wang, Y. Zhu, L. Fan, O. Bastani, D. Jayaraman, DrEureka: Language Model Guided Sim-To-Real Transfer. in Proc. Robotics: Science and Systems (RSS), 2024.
[18] T. Strøm-Hansen, M. Thor, L. B. Larsen, E. Baird, P. Manoonpong, Distributed Sensor-Driven Control for Bio-Inspired Walking and Ball Rolling of a Dung Beetle-Like Robot, in Proc. 2nd International Symposium on Swarm Behavior and Bio-Inspired Robotics (SWARM), 2017, 212.
[19] B. Leung, M. Thor, P. Manoonpong, Modular Neural Control for Bio-Inspired Walking and Ball Rolling of a Dung Beetle-Like Robot, in Proc. Artificial Life Conference (ALIFE), MIT Press, 2018, 335.

[20] M. Thor, T. Strøm-Hansen, L. B. Larsen, A. Kovalev, S. N. Gorb, E. Baird, P. Manoonpong, Artif. Life Robot. 2018, 23, 435.
[21] S. S. Bidaye, T. Bockemuhl, A. Buschges, J. Neurophysiol. 2018, 119, 459.
[22] P. Manoonpong, U. Parlitz, F. Wörgötter, Front. Neural Circuits 2013, 7, 12.
[23] J. Schröder-Schetelig, P. Manoonpong, F. Wörgötter, Auton. Robots 2010, 29, 357.
[24] B. Leung, P. Billeschou, P. Manoonpong, IEEE Trans. Cybern. 2023, 54, 2062.
[25] G. Clifton, A. Y. Stark, C. Li, N. Gravish, J. Exp. Biol. 226, jeb245261 (2023).
[26] Characterizing Terrain Slope and Roughness, http://www.innovativegis.com/basis/MapAnalysis/Topic11/Topic11.htm, accessed: September 2024.
[27] Bijma, N. N., Filonenko, K., Gorb, S. N., Duggen, L., J. Exp. Biol. 2024, 227, jeb245920.
[28] Bijma, N. N., Filippov, A. E., Gorb, S. N., J. Theor. Biol. 2021, 520, 110659.
